# Distribution of Voltage-Gated Sodium Channel (*Nav*) Alleles among the *Aedes aegypti* Populations In Central Java Province and Its Association with Resistance to Pyrethroid Insecticides

**DOI:** 10.1371/journal.pone.0150577

**Published:** 2016-03-03

**Authors:** Sayono Sayono, Anggie Puspa Nur Hidayati, Sukmal Fahri, Didik Sumanto, Edi Dharmana, Suharyo Hadisaputro, Puji Budi Setia Asih, Din Syafruddin

**Affiliations:** 1 University of Muhammadiyah Semarang, Semarang, Indonesia; 2 Graduate School in Medicine and Health, Faculty of Medicine, University of Diponegoro, Semarang, Indonesia; 3 Eijkman Institute for Molecular Biology, Ministry of Research and Technology of the Republic of Indonesia, Jakarta, Indonesia; 4 Health Polytechnic, Jambi Provincial Health Office, Ministry of Health of the Republic of Indonesia, Jambi, Indonesia; 5 Department of Parasitology, Faculty of Medicine, Hasanuddin University, Makassar, Indonesia; University of Queensland & CSIRO Biosecurity Flagship, AUSTRALIA

## Abstract

The emergence of insecticide resistant *Aedes aegypti* mosquitoes has hampered dengue control efforts. WHO susceptibility tests, using several pyrethroid compounds, were conducted on *Ae*. *aegypti* larvae that were collected and raised to adulthood from Semarang, Surakarta, Kudus and Jepara in Java. The *AaNa*_*V*_ gene fragment encompassing kdr polymorphic sites from both susceptible and resistant mosquitoes was amplified, and polymorphisms were associated with the resistant phenotype. The insecticide susceptibility tests demonstrated *Ae*, *aegypti* resistance to the pyrethroids, with mortality rates ranging from 1.6%–15.2%. Three non-synonymous polymorphisms (S989P, V1016G and F1534C) and one synonymous polymorphism (codon 982) were detected in the *AaNa*_*V*_ gene. Eight *AaNa*_*V*_ alleles were observed in specimens from Central Java. Allele 3 (SGF) and allele 7 (PGF) represent the most common alleles found and demonstrated strong associations with resistance to pyrethroids (OR = 2.75, CI: 0.97–7.8 and OR = 7.37, CI: 2.4–22.5, respectively). This is the first report of 8 *Ae*. *aegypti AaNa*_*V*_ alleles, and it indicates the development of resistance in *Ae*. *aegypti* in response to pyrethroid insecticide-based selective pressure. These findings strongly suggest the need for an appropriate integrated use of insecticides in the region. The 989P, 1016G and 1534C polymorphisms in the *AaNa*_*V*_ gene are potentially valuable molecular markers for pyrethroid insecticide resistance monitoring.

## Introduction

Dengue is a growing global problem, with over 3.97 billion people in 128 countries at risk of the disease. Recent estimates number the apparent and inapparent infections in 2010 at approximately 390 million cases [1]. In Indonesia, dengue fever has been reported in all provinces, with an incidence rate (IR) in 2010 reaching 65.7/100.000. Approximately 80.4% of districts/municipalities in Indonesia have reported dengue infections [2]. The three provinces that show the highest dengue incidence include East Java, West Java and Central Java. The number of new dengue cases in Central Java was 19.871 infections in 2010, with an IR of 60.46/100.000 and a case fatality rate (CFR) of 1.26% [2, 3].

Currently, dengue prevention entirely relies on vector control and focuses on routine larval source management and reactive space spraying or other adult-focused campaigns [4]. Unfortunately, the emergence of insecticide resistance in *Aedes aegypti* and the lack of an efficacious dengue vaccine have hampered large-scale efforts for dengue control. This vector has been found to be resistant to various pyrethroid insecticides such as α-cypermethrin in Brazil [5–7], permethrin in Thailand [8] and Mexico [9], and deltamethrin and α-cypermethrin in Thailand [10]. Reports from Indonesia indicate that *Ae*. *aegypti* mosquitoes are resistant to pyrethroid insecticides such as deltamethrin and permethrin in Bandung, Palembang, Surabaya [11] and Semarang [12], whereas resistance to α-cypermethrin has been reported in Central Kalimantan [2].

Molecular studies over the last few decades have identified several mosquito genes and large enzyme families that are involved in insecticide resistance. [13]. One of the most common target site mechanisms that confers resistance to pyrethroid insecticides and DDT is linked to single nucleotide polymorphisms (SNPs) on the Voltage-gated sodium channel (VGSC), collectively referred to as knock down resistance (kdr) alleles. Resistance to organophosphate insecticides is associated with single nucleotide polymorphisms in the acetylcholinesterase-1 (*ace-1*) gene that produces an amino acid change in the encoded acetylcholinesterase (AChE) enzyme. These mutations include G119S, F290V [14, 15] and F455W [16] but so far have never been reported in *Ae*. *aegypti*. Studies have also identified several polymorphisms in genes encoding detoxifying enzymes of the cytochrome P450 monooxygenases, esterases and GSH-S transferase [13]. Overexpression of several subunits of the CYP450 enzymes are associated with resistance to pyrethroid insecticides in anophelines and culicine mosquitoes [17, 18,19,20]. In *Ae*. *aegypti* particularly, several subunits of the *Aedes* P450 such as CYP9J32, CYP9J24 and CYP9J28 have been associated with resistance to pyrethroids [21,22].

To date, several SNPs in the *Aedes aegypti* Natrium voltage-gated channel (*AaNa*_*V*_^)^ gene have resulted in amino acid changes in the sodium channel protein, including L982W [12], S989P [23], I1011M/V [7, 12, 24], L1014F/S [25, 26], V1016G/I [12, 24, 27], and F1534C [28, 29]. These changes have been documented in the anopheline and culicine mosquito populations across wide geographic regions of the world. Different from anophelines and other insects, the mutations in *AaNa*_*V*_ that confer resistance to the pyrethroids in *Aedes aegypti* are S989P [23], I1011M, 1016G/I [7, 9, 24, 28, 30], and F1534C [28, 29, 31]. This study aims to determine the *AaNa*_*V*_ haplotype frequencies, based on the existence of the kdr alleles, among *Aedes aegypti* populations and their association with resistance to pyrethroid insecticides in the dengue endemic areas of Central Java Province, Indonesia. The history of insecticide use in the area was also evaluated through secondary data collection.

## Materials and Methods

### Study site and history of insecticide

**Fig 1:**
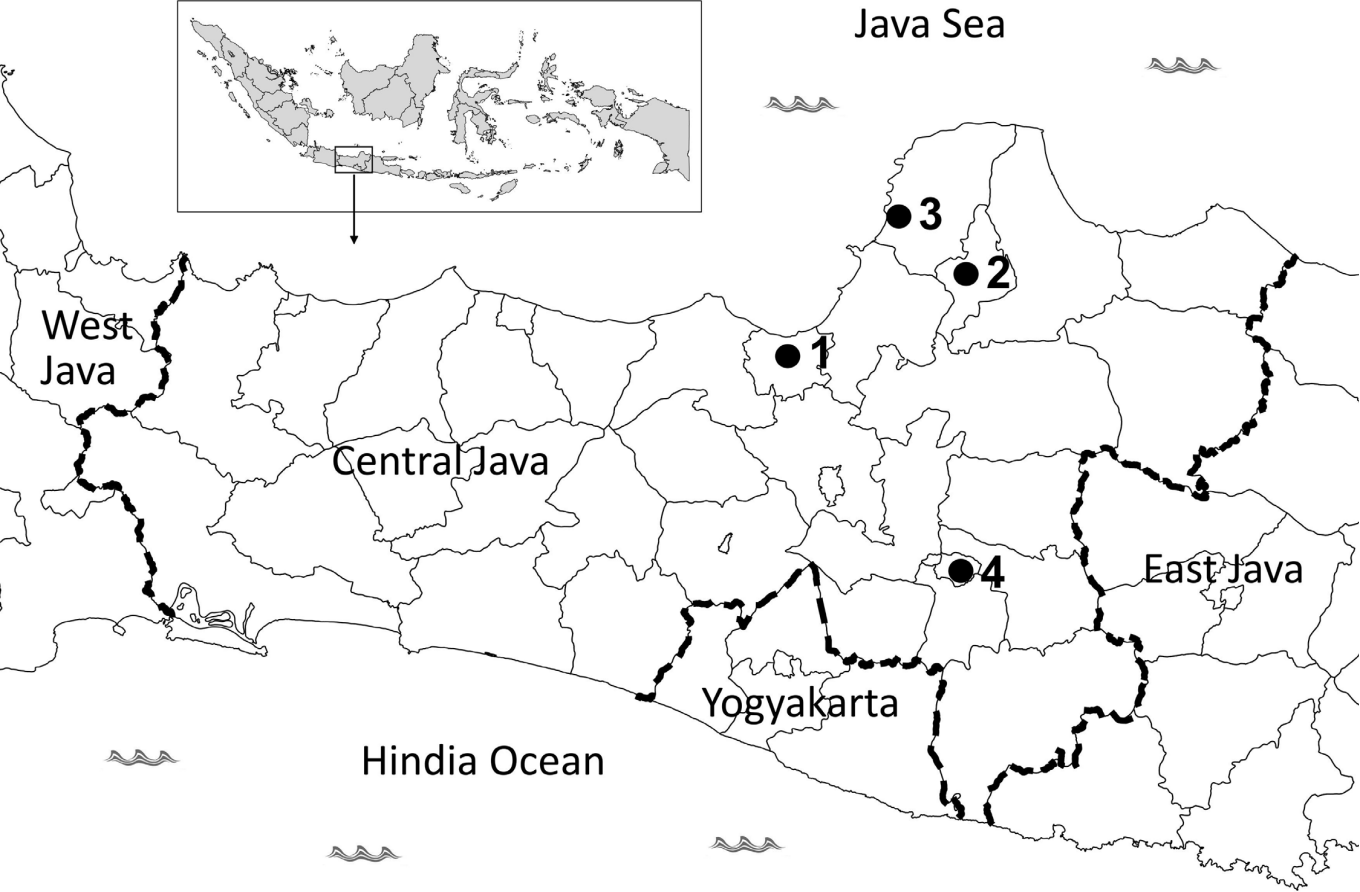
A sketch map of the Central Java Province indicating the location of the study site within the archipelago of Indonesia (insert). The study was conducted in (1) Semarang municipality, (2) Kudus district, (3) Jepara district, and (4) Surakarta municipality.

The study was conducted in 2 districts and 2 municipalities in Central Java Province, Indonesia. Semarang municipality and Jepara district are located on the northern coast of Java island, whereas the Surakarta municipality and Kudus district are located inland (Fig 1). The history of insecticide use was obtained through recorded data at the provincial and district/municipality Health Departments and through direct interviews with household residents where larval collections were performed. Before conducting the study, we submitted the research procedures to the ethics committee at the Faculty of Medicine, Diponegoro University, which recommended that we seek approval only from the Health Department and the households, provided that the procedure does not introduce any potential physical and psychological risks to the subjects (household).

### Mosquito collection

*Aedes aegypti* larvae were collected from 12 household clusters in four dengue endemic areas in Central Java (the Semarang and Surakarta municipalities and the Kudus and Jepara districts) through case-driven cross sectional surveys from January to July 2012. Household clusters were determined using confirmed dengue-positive houses as a core with neighboring houses within a distance of 100 m included in the cluster. The global positioning system (GPS) coordinates of the household cluster is shown in S1 Table. Each cluster consisted of 10–19 households, and the larvae were collected using a dipper. The larval sites were mostly water containers located in- and outside the houses. The larvae were reared to the adult stage (3–5 days old) in a laboratory insectary and were subjected to WHO susceptibility tests using nitrocellulose paper impregnated with one of several pyrethroid or organophosphate compounds. Permission to conduct mosquito and larval collection in the households was given by the provincial and district/municipality authorities following recommendations by the District/Municipalities Health Department.

### Insecticide susceptibility tests

Insecticide susceptibility tests were performed using standard WHO bioassay procedures with diagnostic test kits and impregnated papers [32]. The test papers included 0.05% α-cypermethrin, 0.05% deltamethrin, 0.05% λ-cyhalothrin and 0.8% malathion to assess cross-resistance to these compounds. In each test group, 125 female mosquitoes from each cluster were divided into 5 tubes, and each tube contained 25 adults. Four replicates of each treatment compound were performed in conjunction with a matched control. The test was repeated three times for each insecticide, as recommended by the referenced WHO protocol. Temperature and humidity during the test and holding were maintained at ranges of 25±2°C and 80%±10%, respectively. The following day, the numbers of dead mosquitoes were calculated to determine the resistance status. Resistance criteria of mosquitoes were as follows: resistant if the mortality was <90%, tolerant if the mortality was 90%-98%, and susceptible if the mortality was >98%. The resistance level criteria were RR95 <5 = low, <10 = moderate, <50 = high, <1000 = very high [29], and >1000 = super resistant [33]. Resistance ratios of the mosquitoes to the insecticide were determined using the probit analysis as described previously [7, 23]. The control susceptible strain (entomology group of The National Atomic Energy Agency, Indonesia) originated from Jakarta local *Aedes aegypti* mosquitoes and has been in colony since 1980. The RR95 was calculated by comparing the KDT95 of the wild strains with the KDT95 of the controls. Insecticide resistance tests evaluating the RR95 were focused on α-cypermethrin, the most commonly used compound in Java.

### DNA extraction and PCR amplification

Mosquito thoracal and abdominal parts were homogenized individually in 1.5 ml Eppendorf microtubes containing 50 μl of grinding buffer (0.1 M NaCl, 0.2 M sucrose, 0.1 M Tris HCl, 0.05 M EDTA, and 0.5% sodium dodecyl sulfate (SDS); pH adjusted to 9.2). The genomic DNA was extracted using a Chelex-100 ion exchanger, following the procedure previously described [26]. Fragments of the *AaNa*_*V*_ gene encompassing domain IIS6 and domain IIIS6 with a predicted length of 473 bp and 350 bp, respectively, were amplified separately using a single-step polymerase chain reaction (PCR) with specific primers [24,28] (AaNavF20_kdr): 5’-ACAATGTGGATCGCTTCCC-3'-(AaNavR21_kdr): 5'-TGGACAAAAGCAAGGCTAAG-3' and (AaNavEx31P): 5’-TCGCGGGAGGTAAGTTATTG-3’-(AaNavEx31Q): 5’- GTTGATGTGCGATGGAAATG-3’. The PCR reaction mixture consisted of 25 μl containing of 5 μl template DNA; 50 mM KCl; 10 mM Tris-HCl, pH 8.3; 1.5 mM MgCl2; 200 mM dNTP; 1 U Taq Polymerase and a pair of primers (20 pM each). The reactions were performed in a thermocycler for 5 minutes at 95°C for initial denaturation, followed by 40 cycles of 30 s at 95°C for denaturation, 30 s at 58°C for annealing, 30 s at 72°C for elongation and finally 5 minutes at 72°C polymerase extension, according to the KAPA kits instructions (KAPABIOSYSTEMS, Boston, MA, USA). Electrophoresis of 5 μl aliquots of the PCR products in 2.0% agarose gels confirmed amplification. The purified amplicons were sequenced using an ABI PrismTM Dye BigDye Terminator Cycle Sequencing Ready Kit (Applied Biosystem, Foster City, USA) in an automatic sequencer through fluorescent DNA capillary electrophoresis (ABI 3130×l) at the Eijkman Institute, Jakarta, Indonesia. The DNA sequences obtained were analyzed using an alignment editor program (Biological Sequence Alignment Editor, BioEdit, ver 7.0.9, Ibis Biosciences, Carlsbad, CA, USA). The DNA sequencing was performed bidirecttionally to validate the target polymorphic sites. Descriptive and association statistical analyses were performed with SPSS software.

The association between each haplotype with resistance to pyrethroids was assessed using the control susceptible alleles as a comparison.

## Results

### History of insecticide use

Pyrethroid and organophosphate-based insecticides have been used for dengue vector control in Central Java Province since 1999. The pyrethroid compounds included permethrin, deltamethrin, λ-cyhalothrin and α-cypermethrin. Organophosphate compounds included malathion and temephos [3]. Exposure to insecticides, particularly to pyrethroids, in Semarang and Kudus were more frequent (average 3–4 times fogging to counter a dengue epidemic in three year), while in Jepara and Surakarta, the fogging intensity is between 1–2.5 times (Table 1). The use of household insecticides, such as mosquito coils or sprays, was also common throughout the surveyed area. Surveillance of 159 households in the study area revealed that more than half of the households used either mosquito coils or sprays to protect themselves from mosquito bites almost every day (Table 1). All mosquito coils and sprays utilized pyrethroid compounds.

**Table 1:** Fogging frequency and insecticide used in dengue endemic areas of Central Java for the period of 2010–2012.

Districts/municipalities	Fogging intensity				Households that declared using insecticides	
	Minimum	Maximum	Mean	95% CI	N	% (n)
Semarang	1	8	4.10	3.45–4.75	69	56.5 (39)
Kudus	2	3	2.72	2.53–2.91	25	64.0 (16)
Jepara	1	4	2.54	2.03–3.05	37	48.6 (18)
Surakarta	1	1	1.00	-	28	57.1 (16)
Total	1	8	2.97	2.62–3.33	159	56.0 (89)

### Insecticide susceptibility tests

Insecticide susceptibility tests against the pyrethroid compounds revealed different mortality rates depending on the geographic location (Table 2). Exposures to λ -cyhalothrin rendered mortality rates ranging from 1.3%-37.3% (mean = 20.6%), whereas for deltamethrin, the mortality ranged from 9.3%-86.7% (mean = 27.6%). Exposure to 0.05% α-cypermethrin yielded mortality rates ranging from 1.6% to 15.2%, with an average of 7.92% and a 95% confidence interval (CI) of 4.297 to 11.537. The α-cypermethrin resistance ratio was 95% (RR_95_) 10.4–303.8 times (mean = 69.97 and 95% CI = 14.5–125.5). Mortality rates and resistance levels did not differ by district/municipality (F: 0.342 and 0.687 and p = 0.796 and 0.585, respectively). The RR_95_ values suggest that resistance to alphacypermethrin in *Aedes aegypti* from these surveyed areas was high with all mosquitoes showing a RR_95_>10) and 33.3% of those shwing a RR_95_>50 (Table 3). Exposure to the organophosphate compound malathion yielded mortality rates ranging from 1.3%-85.3% (mean 45.1%), and in several sites, the *Ae*. *aegypti* exhibited tolerance to this chemical.

**Table 2:** Mortality rates of mosquitoes in the susceptibility test using the WHO standard bioassay.

Study sites		24 hours mortality rates of tested-mosquitoes				RR95 to
Districts/municipalities	Villages	α-cypermethrin	Deltamethrin	λ-cyhalothrin	Malathion	α-cypermethrin
Semarang	Jomblang	12.0	29.3	37.3	85.3	22.7
	Kedungmundu	1.6	9.3	1.3	4.0	126.9
	Sampangan	10.4	33.3	37.3	14.7	25.6
	Sendangguwo	2.4	10.7	6.7	1.3	303.8
Kudus	Jati Wetan	1.6	13.3	6.7	18.7	140.2
	Pasuruhan Lor	2.4	32.0	14.7	82.7	37.3
	Tanjung	15.2	25.3	30.7	73.3	10.6
Jepara	Jobokuto	8.8	29.3	25.3	57.3	22.6
	Kuwasen	15.2	22.7	29.3	38.7	10.4
	Pengkol	1.6	9.3	6.7	54.7	102.2
Surakarta	Ngoresan	8.0	86.7	25.3	32.0	26.7
	Gulon	15.2	29.3	25.3	78.7	10.7
Mean		7.9	27.5	20.6	45.1	70.0
95%CI		4.22–11.52	14.35–40.73	12.48–28.71	25.35–64.87	14.5–125.5

**Table 3:** Genotypic pattern in *AaNa*_*V*_ gene of *Ae*. *aegypti* from Central Java.

Districts/municipalities	KDR	SNPs in *AaNa*_*V*_ gene				Number of
	Phenotypes	IIS6 Region			IIIS6 Region	Mosquitoes
		TTG(L)-982-TTA	TCC(S)-989-CCC(P)	GTA(V)-1016-GGA(G)	TTC(F)-1534-TGC (C)	
		TTA	TCC	GGA	TTC	7
		TTA	TCC/CCC	GGA	TTC	5
	Sensitive	TTA	TCC	GTA	TTC	4
	(19)	TTA	TCC	GGA	TTC/TGC	2
		TTA	TCC	GTA/GGA	TTC	1
Semarang						
	Resistant	TTA	TCC	GGA	TTC	11
	(20)	TTA	CCC	GGA	TTC	5
		TTA	TCC/CCC	GGA	TTC	4
	Sensitive	TTA	TCC	GGA	TTC	14
	(17)	TTA	TCC/CCC	GGA	TTC	2
		TTG/TTA	TCC	GGA	TTC	1
Kudus						
		TTA	CCC	GGA	TTC	4
	Resistant	TTA	TCC/CCC	GGA	TTC	3
	(11)	TTA	TCC	GGA	TTC	3
		TTG	TCC	GTA	TTC	1
		TTA	TTC	GGA	TTC	7
	Sensitive	TTA	TCC/CCC	GGA	TTC	7
	(19)	TTA	CCC	GGA	TTC/TGC	2
Jepara		TTA	TCC	GTA/GGA	TTC/TGC	2
		TTA	TCC	GGA	TTC/TGC	1
		TTA	TCC	GGA	TTC	7
	Resistant	TTA	TCC/CCC	GGA	TTC	3
	(14)	TTA	CCC	GGA	TTC	2
		TTA	TCC	GTA	TTC	1
		TTA	TCC/CCC	GTA	TTC/TGC	1
		TTA	TCC	GGA	TTC	9
		TTA	TCC/CCC	GGA	TTC	5
	Sensitive	TTG	TCC	GTA	TTC/TGC	3
	(20)	TTG	CCC	GTA	TTC	1
		TTA	CCC	GGA	TTC	1
		TTA	TCC	GGA	TTC/TGC	1
Surakarta						
		TTA	TCC	GGA	TTC	13
	Resistant	TTA	TCC/CCC	GGA	TTC	7
	(27)	TTA	CCC	GGA	TTC	6
		TTA	TCC	GGA	TGC	1
Total	147					147

Note: IIS6 region: 982L(TTG/TTA)+S(TCC)989P(CCC)+V(GTA)1016G(GGA).

IIIS6 region: F(TTC)1534C(TGC). The phenotype was based to sensitivity to α—cypermethrin only.

### Molecular analysis

Molecular analyses of 147 *Ae*. *aegypti* isolates from Central Java Province, Indonesia revealed the existence of SNPs at codons 982L (TTG→TTA), S989P (TCC→CCC), V1016G (GTA→GGA) of the IIS6 region and F1534C (TTC→TGC) of the IIIS6 region in the *AaNa*_*V*_ gene (Table 3). Codon numeration was adopted from the published sequence of the *Musca domestica Na*_*V*_ gene (Genbank ID: KJ957878-KJ957893). Synonymous nucleotide substitutions that lead to a silent mutation (TTG to TTA) at codon 982L predominated in all of the mosquito isolates examined (96.2%). A nucleotide substitution that led to an amino acid change from V to G at codon 1016 was found in 91.2% of the samples. A nucleotide substitution that lead to an amino acid change S to P at codon 989 was found in 25% of the isolates, either as a single mutation in the *AaNa*_*V*_ gene or in combination with mutation V1016G. No polymorphisms were found in the IIS6 intron region. A nucleotide substitution, at codon 1534, that lead to an F to C amino acid change was observed less frequently (3%) in three of the surveyed areas (except the Kudus District) and appeared either as a single *AaNa*_*V*_ mutation or in combination with the other mutations in the IIS6 regions. Overall, four haplotypes; 989S+1016V, 989S+1016G, 989P+1016V and 989P+1016G were observed in the IIS6 region, and 2 haplotypes; 1534F and 1534C were observed in the IIIS6 region (Table 3).

### Allele frequency and association with the resistance to pyrethroids

Eight alleles of the S989P, V1016G and F1534C SNPs were identified in *Aedes aegypti* isolates with different frequency distributions (Table 4). Allele 1 represented the wildtype allele that was found in the control *Ae*. *aegypti* originating from laboratory colonies and in a small proportion of the isolates from field collected mosquitoes (7.1%). Alleles 2 and 5 carried single *Na*_*V*_ polymorphism, 1534C and 989P, respectively. The most common *AaNa*_*V*_ allele was allele 3, which carried a single *Na*_*V*_ polymorphism, 1016G (62.6%). Allele 7 carried double *Na*_*V*_ polymorphisms, 989P/1016G, and constituted 25.8% of the samples. Allele 4 carried double *Na*_*V*_ polymorphisms, 1016G/1534C (2%), and allele 6 carried double *Na*_*V*_ polymorphisms, 989P/1534C (0.3%). Allele 8 carried triple *Na*_*V*_ polymorphisms, 989P/1016G/1534C, and was found in one isolate.

**Table 4:** The *AaNa*_*V*_VGSC alleles and their association with resistance to α—cypermethrin.

Alleles	Phenotypes		Total	OR
	Sensitive	Resistant		
Allele 1 (SVF)	16^+^ (76.2*)	5 (23.8*)	21(7.1)	-
Allele 2 (SV**C**)	3 (100)	0 (0.0)	3(1)	1.07
Allele 3 (S**G**F)	99 (53.8)	85 (46.2)	184(62.6)	2.75 (0.97 to 7.8)
Allele 4 (S**GC**)	4 (66.7)	2 (33.3)	6(2)	1.6 (0.22 to 11.5)
Allele 5 (**P**VF)	2 (100)	0 (0.0)	2(0.7)	1.60
Allele 6 (**P**V**C**)	0 (0.0)	1 (100)	1(0.3)	3.20
Allele 7 (**PG**F)	23 (30.3)	53 (69.7)	76(25.8)	7.37 (2.4 to 22.5)
Allele 8 (**PGC**)	1 (100)	0 (0.0)	1(0.3)	3.20
Total	148	146	294	

Bold Letter = mutant type [S989**P**. V1016**G**. F1534**C**].

(*) = indicates percentage.

(^+^) = indicates total samples.

OR (odds ratio) value higher than 1 indicates strong association between genotype and phenotype.

A strong association was found between the alleles 3 and 7 with resistance to α- cypermethrin with odds ratios (OR) of 2.75, CI: 0.97–7.8 and 7.37, CI: 2.4–22.5, respectively (Table 4). The remaining alleles occurred at a much lower frequency and were not used for this analysis.

## Discussion

Pyrethroid compounds have increasingly been used in Indonesia within the last 15 years due to their rapid knockdown effect and relatively low hazard to mammalia, in comparison to other insecticides. In Central Java Province, pyrethroids have also been widely used as household insecticides to control mosquitoes and other household insect pests. *Aedes* mosquitoes preferentially live around human dwellings and lay their eggs in the water containers inside and around the houses. Therefore, constant exposure of these mosquitoes to household insecticides is unavoidable, resulting in a rapid selection for resistance to pyrethroids. Although the fogging intensity is different in each site, the resistance status of the *Ae*. *aegypti* in each site is not significantly different. This finding indicates that other means of insecticide exposure may have taken place such as the use of household insecticide, such as spatial repellent.

Insecticide susceptibility tests performed using 3 different pyrethroid compounds (α-cypermethrin, λ-cyhalothrin and deltamethrin) revealed high levels of resistance in *Ae*. *aegypti* populations from 4 different localities in Central Java Province, Indonesia. The findings are consistent with previous reports for two of the surveyed areas where *Ae*. *aegypti* samples were collected–Semarang and Salatiga municipalities. The previous surveys reported that *Aedes aegypti* isolates were resistant to permethrin, with a resistance level ranging from moderate to high [12]. The current study involved a wider geographic region of Central Java Province, and the findings also demonstrated a significant shift of the *Ae*. *aegypti* population to a higher pyrethroid resistance level. Several reasons may explain this significant increase: First, the pyrethroid compounds have been continuously used in the surveyed areas, either through fogging activities or insecticide residual spray (IRS), to control adult mosquitoes. Secondly, household insecticides and spatial repellents that possess active pyrethroid ingredients are being more widely used to control the mosquitoes and other household insect pest populations and therefore maintain the constant exposure of the *Ae*. *aegypti* population to the pyrethroids.

Molecular analyses of samples in this study revealed three *AaNa*_*V*_ gene mutations that led to mutated amino acid residues at 989P, 1016G and 1534C among the *Ae*. *aegypti* population. The 1016G and 1534C mutation have been widely reported and are tighly linked to the resistance to type I pyrethroids, permethrin [12, 20,23, 24, 25]. The 989P mutation was initially reported among the laboratory colony of *Ae*. *aegypti* after selection with the type II pyrethroid, deltamethrin and later was found among the field collected permethrin resistant *Ae*. *Aegypti* isolates in Thailand [19]. Its association with pyrethroid resistance, however, still has to be confirmed. In this study, resistance to α-cypermethrin, a type I pyrethroids exhibited 8 different *AaNa*_*V*_ alleles. Allele 1 (SVF) represents the wildtype allele that carried none of the *Na*_*V*_ polymorphism, and it was found in the laboratory colony and in a small proportion of the samples that show resistance to α- cypermethrin. The finding suggests the existence of other mechanisms, such as metabolic resistance and resistance status to organophosphates. In this regard, it is of particular interest to examine the role of the detoxifyng enzymes such as subunits of the P450 enzymes in this mosquito samples [21,22].

Alleles 2, 3 and 5 each carried one of the *Na*_*V*_ polymorphism, 989P, 1016G or 1534C. Allele 3, which carried a single *Na*_*V*_ polymorphism, 1016G, is the most common allele found in this study. This finding supports the previous notion that mutation at codon 1016 appears to be the most common *AaNa*_*V*_ gene mutation in *Ae*. *aegypti* reported worldwide and is associated with resistance to pyrethroids [12, 23, 27, 34, 35, 36]. However, recent evidence indicates that in Latin America, mutation in codon 1534 is more common (28. 31, 37, 38). The 1016G mutation, so far, has never been identified from Latin America, and instead, the 1016I mutation predominates in that region. While it can contribute to permethrin resistance in Latin America, other mechanisms may also play role [9, 37, 38]. The frequency distribution of the 1016G mutation among *Ae*. *aegypti* populations in Central Java Province has almost doubled in the last 10 years, when compared to previous reports [12]. Despite very limited data on the frequency distribution of kdr alleles among the *Ae*. *aegypti* populations in Indonesia, the rapid increase in frequency and distribution of this allele might be explained by a constant positive pressure by pyrethroid compounds, similar to what occurred in the 1016I allele in Mexico [9]. Allele 5, characterized by a single *Na*_*V*_ polymorphism, 989P, was first reported in Indonesia and was previously associated with resistance to deltamethrin in a laboratory selected *Ae*. *aegypti* strain in addition to field collected mosquitoes [23, 34, 35]. The existence of this haplotype among mosquito populations in this study is understandable, as deltamethrin has been used extensively in the study area, although α-cypermethrin is currently more widely used.

Alleles carrying the 1534C mutation occurred at a lower frequency in Central Java in comparison to Thailand, where it was linked to permethrin resistance [34–36]. The difference in this frequency distribution of the *Na*_*V*_ allele in various geographic regions around the world might be explained by different pyrethroid compounds used in each region. The existence of the 1016G mutation in Thailand was associated with resistance to the type II pyrethroid deltamethrin. The other explanation is that the origin of the *Ae*. *aegypti* natural population in each localities might be different as was shown previously in Brazil [37]. Unfortunately, until now very limited information is available on the genetic origin of the *Ae*. *aegypti* population in any localities in Indonesia. Alleles carrying the double or triple *Na*_*V*_ polymorphisms 989P/1016G/1534C were associated with stronger resistance to α-cypermethrin [31]. In this study, due to a small sample size, only allele 7, which carried double *Na*_*V*_ allele, 989P/1016G, was found to be strongly associate with pyrethroid resistance. This finding is supported by previous reports in which the individual mosquitoes carrying the same 989P/1016G *Na*_*V*_ polymorphisms in the presence of homozygous 1534F polymorphism survived exposure to permethrin [35]. Although the alleles carrying double or triple *Na*_*V*_ polymorphisms occurred at a much lower frequency, the findings suggest a need for a resistance management plan at the study site. Previous studies have indicated that the co-existence of *Na*_*V*_ polymorphisms 1016I and 1534C is tighly linked to the loss of susceptibility to pyrethroids [12, 38]. The 1534C mutation was first reported in Indonesia and was found to co-exist in a homozygous status with the 1016G mutation in one mosquito sample examined. Regular monitoring is required to evaluate and understand population level dynamics of insecticide resistance alleles in Indonesia and their association with resistance to pyrethroids. These findings also suggest the advantage of using alternative insecticides in areas where *Ae*. *aegypti* mosquitoes are pyrethroid resistant. Consequently, the proper use of organophosphate, carbamates and insect growth regulator–for biological control of both the adult and larval stages of the mosquitoes may be considered. However, the insecticide susceptibility tests also showed a degree of the *Ae*. *aegypti* resistance to organophosphate compound in certain area in Central Java. As the molecular analyses on the *ace1* gene of the resistant mosquitoes revealed that all still carried the wildtype allele, resistance to organophosphate in the area might be related to metabolic resistance (Data will be published elsewhere).

## Conclusions

*Ae*. *aegypti* in dengue endemic areas of Central Java Province is highly resistant to pyrethroid insecticides, and 8 *Na*_*V*_ alleles that carried the 989P, 1016G and 1534C *Na*_*V*_ polymorphisms, either alone or in combination, were revealed. The overall findings strongly encourage the regular monitoring of insecticide resistance and appropriate insecticide selection and use for the resistance phenomenon to be contained.

## Supporting Information

S1 Fig Knockdown assay results in *Aedes aegypti*. (DOC)

S1 Table Mosquito collection sites and its GPS coordinates. (DOC)

## References

[1] Bhatt S, Gething PW, Brady OJ, Messina JP, Farlow AW, Moyes CL, et al The global distribution and burden of dengue. Nature. 2013; 496 (7446):504–507. 10.1038/nature12060 23563266 PMC3651993

[2] Brahim R, Sitohang V, Zulkarnaen I. Profil Kesehatan Indonesia 2010. Jakarta: Kementerian Kesehatan Republik Indonesia 2011.

[3] Dinas Kesehatan Provinsi JawaTengah. Data Kasus DBD di Jawa Tengah Tahun 2010. In.: Dinas Kesehatan Provinsi Jawa Tengah 2010.

[4] Achee NL, Gould F, Perkins TA, Reiner RC Jr, Morrison AC, Ritchie SA, et al A critical assessment of Vector control for dengue prevention. PloS Negl Trop Dis. 2015; 9(5). 10.1371/journal.pntd.0003655 PMC4423954 25951103

[5] Luna JED, Martins MF, Anjos AFd, Kuwabara EF, Navarro-Silva eMA. Susceptibility of *Aedes aegypti* to temephos and cypermethrin insecticide, Brazil. Rev Saude Publica. 2004; 38(6):1–2. 15608903 10.1590/s0034-89102004000600013

[6] da-Cunha MP, Lima JBP, Brogdon WG, Moya GE, Valle D. Monitoring of resistance to the pyrethroid cypermethrin in Brazilian *Aedes aegypti* (Diptera: Culicidae) populations collected between 2001 and 2003. Mem Inst Oswaldo Cruz. 2005; 100(4):441–444. 16113895 10.1590/s0074-02762005000400017

[7] Lima EP, Paiva MHS, Araujo APd, Silva EVGd, Silva UMd, Oliviera LNd, et al Insecticide resistance in *Aedes aegypti* populations from Ceara, Brazil. Parasites Vectors. 2011; 4(1):5. 21226942 10.1186/1756-3305-4-5 PMC3035027

[8] Ponlawat A, Scott JG, Harrington LC. Insecticide Susceptibility of *Aedes aegypti* and Aedes albopictus across Thailand. J Med Entomol. 2005; 42(5):821–825. 16363166 10.1603/0022-2585(2005)042[0821:ISOAAA]2.0.CO;2

[9] Garcia GP, Flores AE, Fernandez-Salas I, Saavedra-Rodriquez K, Reyes-Solis G, Lozano-Fuentes S, et al Recent Rapid Rise of Permethrin Knock Down Resistance Allele in *Aedes aegypti* in Mexico. PLos Neg Trop Dis. 2009; 3(10). 10.1371/journal.pntd.0000531 PMC2759509 19829709

[10] Thanispong K, Sathantriphop S, Chareonviriyaphap T. Insecticide resistance of *Aedes aegypti* and *Culex quinquefasciatus* in Thailand. J Pestic Sci. 2008; 33(4):351–356.

[11] Ahmad I, Astari S, Tan M. Resistance of Aedes aegypti (Diptera: Culicidae) in 2006 to Pyrethroid Insecticides in Indonesia and its association with Oxidase and Esterase Levels. Pakistan J Biol Sci. 2007; 10(20):3688–3692. 10.3923/pjbs.2007.3688.3692 19093483

[12] Brengues C, Hawkes NJ, Chandre F, McCarroll L, Duchon S, Guillet P, et al Pyrethroid and DDT cross-resistance in *Aedes aegypti* is correlated with novel mutations in the voltage-gated sodium channel gene. Med Vet Entomol. 2003; 17:87–94. 12680930 10.1046/j.1365-2915.2003.00412.x

[13] Hemingway J, Ranson H. Insecticide Resistance in Insect Vectors of Human Diseases. Annu Rev Entomol. 2000; 45:371–391. 10761582 10.1146/annurev.ento.45.1.371

[14] Osta MA, Rizk ZJ, Labbe P, Weill M, Knio K. Insecticide resistance to organophosphates in *Culex pipiens* complex from Lebanon. Parasites Vectors. 2012; 5(132):1–6. 22759898 10.1186/1756-3305-5-132 PMC3414835

[15] Alou LPA, Koffi AA, Adja MA, Tia E, Kouassi PK, Kone M, et al Distribution of ace-1R and resistance to carbamates and organophosphates in *Anopheles gambiae* s.s. populations from Côte d'Ivoire. Malar J. 2010; 9(167). 10.1186/1475-2875-9-167 PMC2908637 20553593

[16] Nabeshima T, Mori A, Kozaki T, Iwata Y, Hidoh O, Harada S, et al An amino acid substitution attributable to insecticide-insensitivity of acetylcholinesterase in a Japanese encephalitis vector mosquito, *Culex tritaeniorhynchus*. Biochem Biophys Res Com. 2004; 313:794–801. 14697262 10.1016/j.bbrc.2003.11.141

[17] Komagata O, Kasai S, Tomita T. Overexpression of cytochrome P450 genes in pyrethroid-resistant *Culex quinquefasciatus*. Insect Biochem Molec. 2010; 40:146–152. 10.1016/j.ibmb.2010.01.006 20080182

[18] Itokawa K, Komagata O, Kasai S, Okamura Y, Masada M, Tomita T. Genomic structures of Cyp9m10 in pyrethroid resistant and susceptible strains of *Culex quinquefasciatus*. Insect Biochem Molec. 2010; 40(9):631–640. 10.1016/j.ibmb.2010.06.001 20600899

[19] Saavedra-Rodriguez K, Strode C, Flores AE, Garcia-Luna S, Reyes-Solis G, Ranson H, et al Differential transcription profiles in *Aedes aegypti* detoxification genes following temephos selection. Insect Mol Biol. 2014; 23 (2):199–215. 10.1111/imb.12073 24299217 PMC4091897

[20] Faucon F, Dusfour I, Gaude T, Navratil V, Boyer F, Chandre F, et al Unravelling genomic changes associated with insecticide resistance in the dengue mosquito *Aedes aegypti* by deep targeted sequencing. Genome Res. 2015; 115. 10.1101/gr.189225.115 PMC4561493 26206155

[21] Strode C, Wondji CS, David JP, Hawkes NJ, Lumjuan N, Nelson DR, et al Genomic analysis of detoxification genes in the mosquito *Aedes aegypti*. Insect Biochem. Mol Biol. 2008; 38,113–123. 18070670 10.1016/j.ibmb.2007.09.007

[22] David JP, Ismail HM, Chandor-Proust A, Paine MJI. Role of cytochrome P450 in insecticide resistance: impact on the control of mosquito-borne diseases and use of insecticide on earth. Phil Trans R Soc B. 2012; 368:20120429. 10.1098/rstb.2012.0429 PMC3538419 23297352

[23] Srisawat R, Komalamisra N, Eshita Y, Zheng M, Ono K, Itok TQ, et al Point Mutation in domain II of the voltage-gated sodium channel gene in deltamethrin-resistant *Aedes aegypti* (Diptera: Culicidae). Appl Entomol Zool. 2010; 45(2):275–282.

[24] Martins AJ, Lins RMMdA, Linss JGB, Peixoto AA, Valle D. Voltage-gated Sodium Channel Polymorphism and Metabolic Resistance in Pyrethroid-Resistant *Aedes aegypti* from Brazil. Am J Trop Med Hyg. 2009; 81(1):108–115. 19556575

[25] Singh OP, Dykes CL, Das MK, Pradhan S, Bhatt RM, Agrawal OP, et al Presence of two alternative kdr-like mutations, L1014F and L1014S, and novel mutation, V1010L, in the voltage-gated Na+ Channel of *Anopheles culicifacies* from Orissa, India. Malar J. 2010; 9(1):146. 20509922 10.1186/1475-2875-9-146 PMC2895608

[26] Syafruddin D, Hidayati APN, Asih PBS, Hawley WA, Sukowati S, Lobo NF. Detection of 1014F kdr mutation in four major Anopheline malaria vector in Indonesia. Malar J. 2010; 9(1):315. 21054903 10.1186/1475-2875-9-315 PMC2989330

[27] Kawada H, Higa Y, Komagata O, Kasai S, Tomita T, Yen NT, et al Widespread Distribution of Newly Found Point Mutation in Voltage-gated Sodium Channel in Pyrathroid-Resistant *Aedes aegypti* Populations in Vietnam. PLos Neg Trop Dis. 2009; 3(10). 10.1371/journal.pntd.0000527 PMC2754656 19806205

[28] Harris AF, Rajatileka S, Ranson H. Pyrethroid Resistance in *Aedes aegypti* from Grand Cayman. Am J Trop Med Hyg. 2010; 83(2):277–284. 10.4269/ajtmh.2010.09-0623 20682868 PMC2911171

[29] Kasai S, Ng LC, Lam-Phua SG, Tang CS, Itokawa K, Komagata O, et al First Detection of a Putative Knockdown Resistance Gene in Major Mosquito Vector, *Aedes albopictus*. Jpn J Infect Dis. 2011; 64:217–221. 21617306

[30] Martins AJ, Lima JBP, Peixoto AA, Valle D. Frequency of Val1016Ile mutation in the voltage-gated sodium channel gene of *Aedes aegypti* Brazilian populations. Trop Med Int Health. 2009; 14(11):1351–1355. 10.1111/j.1365-3156.2009.02378.x 19735371

[31] Brito LP, Linss JGB, Lima-Camara TN, Belinato TA, Piexoto AA, Lima JBP, et al Assessing the Effects of *Aedes aegypti* kdr Mutations on Pyrethroid Resistance and Its Fitness Cost. PLos One. 2013; 6(4):1–10. 10.1371/journal.pone.0060878 PMC3620451 23593337

[32] World Health Organisation. Test Procedure for Insecticide Resistance Monitoring in Malaria Vectors mosquitoes. In.: WHO/CDS Geneva 2013;.

[33] Ahmad I. Adaptasi Serangga dan Dampaknya terhadap Kehidupan Manusia. Institute Teknologi Bandung; 2011 pp. 47.

[34] Yanola J, Somboon P, Walton C, Nachaiwieng W, Prapanthadara L. A novel F1552/C1552 point mutation in the *Aedes aegypti* voltage-gated sodium channel gene associated with permethrin resistance. Pestic Biochem Physiol. 2010; 96:127–131.

[35] Yanola J, Somboon P, Walton C, Nachaiwieng W, Somwang P, Prapanthadara L. High-throughput assays for detection of the F1534C mutation in the voltage-gated sodium channel gene in permethrin-resistant *Aedes aegypti* and the distribution of this mutation throughout Thailand. Trop Med Int Health. 2011; 16:501–509. 10.1111/j.1365-3156.2011.02725.x 21342372

[36] Stenhouse SA, Plernsub S, Yanola J, Lumjuan N, Dantrakool A, Choochote W, et al Detection of the V1016G mutation in the voltage-gated sodium channel gene of *Aedes aegypti* (Diptera: Culicidae) by allele-specific PCR assay, and its distribution and effect on deltamethrin resistance in Thailand. Parasites Vectors. 2013; 6(253):1–10. 24059267 10.1186/1756-3305-6-253 PMC3765916

[37] Linss JGB, Brito LP, Garcia GA, Araki AS, Bruno RF, Lima JBP, et al Distribution and dissemination of the Val1016Ile and Phe1534Cys Kdr mutations in *Aedes aegypti* Brazilian natural population. Parasites Vectors. 2014; 7(25):1–11. 24428880 10.1186/1756-3305-7-25 PMC3912884

[38] Saavedra-Rodriquez K, Urdaneta-Marquez L, Rajatileka S, Moulton M, Flores AE, Biset J, et al A mutation in the voltage-gated sodium channel gene associated with pyrethroid resistance in Latin American *Aedes aegypti*. Insect Mol Biol. 2007; 16(6):785–798. 18093007 10.1111/j.1365-2583.2007.00774.x

