# Supplementary material for: Distribution of Voltage-Gated Sodium Channel (Nav) Alleles among the Aedes aegypti Populations In Central Java Province and Its Association with Resistance to Pyrethroid Insecticides
Source: PLoS One. 2016 Mar 3;11(3):e0150577. doi: 10.1371/journal.pone.0150577 (PMC4777534; doi:10.1371/journal.pone.0150577)
Supplement: S1 Fig — (DOC) [file pone.0150577.s001.doc]

Figure S1. Knock downs assay result of the *Aedes aegypti* mosquitoes from different sites in Central Java Province. Following exposure with pyrethroids (α-cypermethrin)


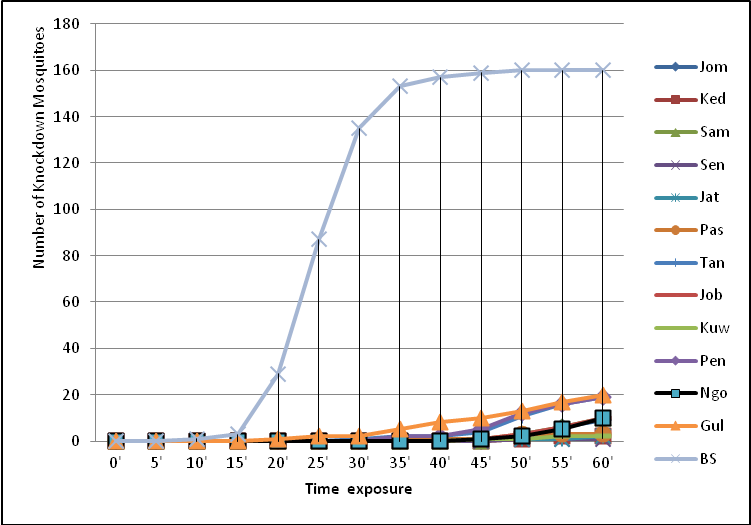


Sites:

Jom = Jomblang

Ked = Kedungmundu

Sam = Sampangan

Sen = Sendangguwo

Jat = Jatiwetan

Pas = Pasuruhan Lor

Tan = Tanjung

Job = Jobokuto

Pen = Pengkol

Kuw = Kuwasen

Ngo = Ngoresan

Gul = Gulon

BS = BATAN Strain (Lab Strain from BATAN)
