# Supplementary material for: Distribution of Voltage-Gated Sodium Channel (Nav) Alleles among the Aedes aegypti Populations In Central Java Province and Its Association with Resistance to Pyrethroid Insecticides
Source: PLoS One. 2016 Mar 3;11(3):e0150577. doi: 10.1371/journal.pone.0150577 (PMC4777534; doi:10.1371/journal.pone.0150577)
Supplement: S1 Table — (DOC) [file pone.0150577.s002.doc]

Table S1. Mosquito collection sites and its GPS coordinates

|  | Mosquito collection sites | GPS coordinate | |
| --- | --- | --- | --- |
|  |  | Latitude | Longitude |
| A | Semarang |  |  |
|  | Jomblang | 7.015724 S | 110.442598 E |
|  | Kedung mundu | 7.022667 S | 110.472920 E |
|  | Sampangan | 7.016576 S | 110.388438 E |
|  | Sendang guwo | 7.011167 S | 110.449076 E |
|  |  |  |  |
| B | Kudus |  |  |
|  | Jatiwetan | 6.84058 S | 110.82385 E |
|  | Pasuruhan Lor | 6.82608 S | 110.81501 E |
|  | Tanjung | 8.84273 S | 110.83475 E |
|  |  |  |  |
| C | Jepara |  |  |
|  | Jobokuto | 6.585271 S | 110.660395 E |
|  | Kuwasen | 6.569966 S | 110.696358 E |
|  | Pengkol | 6.581307 S | 110.688161 E |
|  |  |  |  |
| D | Surakarta |  |  |
|  | Ngoresan | 7.551881 S | 110.855191 E |
|  | Gulon | 7.551562 S | 110.854376 E |
